# Propionate Production by Infant Fecal Microbiota Is Inversely Correlated with the Protein Glycation Level of Supplemented Infant Formula Ex Vivo

**DOI:** 10.3390/nu16234047

**Published:** 2024-11-26

**Authors:** Grégoire A. Bouillon, Zhuqing Xie, Dennis S. Nielsen, Maria Wiese, Arjen Nauta

**Affiliations:** 1 Section for Food Microbiology, Gut Health and Fermentation, Department of Food Science, University of Copenhagen, Rolighedsvej 26, DK-1958 Frederiksberg, Denmarkzhuqing@food.ku.dk (Z.X.);; 2 FrieslandCampina, Stationsplein 4, 3818 LE Amersfoort, The Netherlands

**Keywords:** infant formula, glycation, blocked lysine, microbiota, short-chain fatty acids, propionate

## Abstract

Background/Objectives: After birth, mothers provide the best nutrition for the healthy growth and development of their infants and the developing gut microbiota through breastfeeding. When breastfeeding is not or insufficiently available, infant formula is the only safe alternative. The production of infant formula includes heat-processing, which may induce protein glycation. Protein glycation has been shown to reduce protein digestion and absorption. The reduction in protein digestion and absorption because of protein glycation has been speculated to also impact gut comfort parameters as well as overnight sleep. Methods: As this could be partially due to the effect on the bacteria that reside in the infant’s gastrointestinal tract, we investigated whether protein glycation in infant formula impacts the composition and activity of infant gut microbiota by performing an *in vitro* study using the CoMiniGut colon model and fecal inocula obtained from a healthy six-month-old term infant. Incubations were performed for 24 h using a predigested infant formula-supplemented medium with varying levels of glycation (6.5–44.5%). Results: Our data indicate that high protein glycation increases microbial diversity and the relative abundance of *Clostridium neonatale* from 6.4% of the inoculum to around 25.5% of 20.8% glycation. Interestingly, propionate levels were inversely correlated with protein glycation levels after 24 h of incubation, with the 44.5% blocked lysine sample giving rise to 60% lower propionate levels as compared to the 6.4% sample. Higher propionate levels have been linked with longer uninterrupted sleep overnight, which could be indicative of the underlying mechanism of reduced crying/fussy time during nights for infants fed with a formula containing lower amounts of glycated protein.

## 1. Introduction

After birth, mothers provide the best nutrition for the healthy growth and development of their infants and the developing gut microbiota through breastfeeding. Human milk, as the preferred form of nutrition for infants, contains various valuable macro- and micro-nutrients as well as bioactive components that nourish the newborn but also support the colonization and development of a healthy microbiota in the gastrointestinal tract (GIT) [1,2,3]. When breastfeeding is unavailable or insufficiently available, infant formula is the only safe alternative.

Infants fed with infant formula can suffer from gastrointestinal (GI) complaints, albeit to a different extent depending on the formula [4]. Gut (dis)comfort is affected by a complex combination of parameters such as the composition and the digestibility of (macro-)nutrients [5]. To produce high-quality, nutritious, and easily digestible infant formula, it is crucial to ensure minimal damage to the sensitive (basic) ingredients without jeopardizing microbiological safety [6]. The availability of the essential amino acid lysine is a key indicator determining the final nutritive value of infant formula as it is only bioavailable when its ε-amino group is free [6,7,8,9]. The heat-processing of infant formula triggers a reaction between the ε-amino groups of lysine and lactose, which is called the Maillard reaction or protein glycation [6,10,11]. Glycation makes lysine unavailable for digestion and absorption, resulting in a reduction in lysine bioavailability [12,13]. Therefore, the quantification of “blocked lysine” is a widely used tool to assess the nutritional quality of processed milk products [10,11,14,15].

In addition to the impact of protein glycation on nutritional value, it has also been speculated to negatively affect gut comfort. In a cross-sectional observational study in China, gut comfort parameters between infants exclusively formula-fed with one of four commercially available infant formulas, differing in glycation levels amongst other factors, were assessed and compared [16]. Infants fed with the formula that contained the lowest amount of glycated protein were reported to have less crying/fussy time during the night, and thus a better sleep, and fewer GI symptoms such as flatulence, burping, diarrhea, abdominal distension, and constipation [17]. This could be due to changes in gut microbiota composition and activity, depending on the preferences and/or possibilities of specific intestinal bacteria regarding the metabolization of either intact or glycated proteins [18].

In infants, the establishment of the gut microbiota as thrived by dedicated human milk constituents and the structure and function of the gut as well as the production of digestive enzymes in the gut and pancreas undergo development and maturation [17]. As a result of protein glycation, incompletely digested protein might escape absorption and pass on to the more distal parts of the colon of formula-fed infants, where it may impact the resident microbiota and alter fermentation, yielding undesired products and leading to excessive gas formation, potentially causing GI discomfort [16,19]. To reveal whether differences in protein glycation levels discriminatively impact the composition and activity in infant gut microbiota, an *in vitro* study was performed using the CoMiniGut colon model. Fermentation was carried out in the CoMiniGut for 24 h using a basal medium supplemented with predigested infant formulas with varying levels of glycation (6.5–44.5%) and inoculated using a fecal inoculum from a healthy six-month-old term infant. After fermentation, the production of short- and branched-chain fatty acids was measured through gas chromatography and changes in the microbial community were investigated using 16S rRNA gene amplicon sequencing. Insights into the discriminative impact of protein glycation levels could reveal the underlying mechanisms of the observed differences in sleep and GI symptoms, as observed in the cross-sectional observational study.

## 2. Materials and Methods

### 2.1. Breast Milk

Fresh human milk was collected from two breastfeeding mothers at least 1 month after giving birth. The ages of the infants on the day of human milk collection were 49 and 72 days. On the morning of the *in vitro* gastrointestinal digestion experiment, fresh human milk was collected according to the international donor human milk bank guidelines. Directly following collection, human milk was stored in a sterile thermos flask at ~4 °C and transported to the laboratory. At the laboratory, human milk of the two mothers was pooled and the pooled sample was directly subjected to the *in vitro* gastrointestinal digestion experiment. The pooled human milk sample had a protein concentration of 1.3% and a fat content of 2.1%.

### 2.2. Infant Formula Preparation

Infant formulas with various percentages of blocked lysine (6.5%, 8.4%, 11.8%, 14.8%, 20.8%, and 44.5%) were provided by FrieslandCampina (FrieslandCampina, Amersfoort, The Netherlands). A detailed description of the production of infant formula samples with varying blocked lysine levels is provided by Zenker et al. 2020 [20]. In short, a single batch of infant formula was incubated in a stove at 60 or 70 °C for 0 to 14 days to induce different levels of blocked lysine, and the different levels of lysine blockage were analyzed as previously described [12].

### 2.3. In Vitro Simulated Upper Gastrointestinal Digestion of Infant Formula

The digestion of infant formula and human milk in the stomach and small intestine was simulated through an infant *in vitro* digestion protocol based on that of Menard et al. [21]. This model is the first infant *in vitro* digestion model that was internationally endorsed by INFOGEST and is the most widely used static *in vitro* model internationally. In short, infant formula was dissolved in demineralized water at a standard protein level of 1.6%, as instructed by the manufacturer. The gastric phase was simulated through the addition of simulated gastric fluid (94 mM NaCl, 13 mM KCl, adjusted to pH 5.3 with 1 M HCl) containing porcine pepsin (268 U/mL) and *Rhizopus oryzae* lipase (19 U/mL) enzymes. Infant formula and human milk were incubated with simulated gastric fluid for 60 min at 37 °C and pH 5.3. After 60 min, simulated intestinal fluid (164 mM NaCl, 10 mM KCl, 85 mM NaHCO_3_, 3 mM CaCl) containing porcine pancreatin (16 U/mL trypsin activity) and bovine bile (3.1 mM) was added to simulate the intestinal phase. The samples were incubated for another 120 min at 37 °C and pH 6.6. Samples were taken following 180 min of simulated gastrointestinal digestion (i.e., 60 min gastric; 120 min intestinal) and enzyme activity was stopped via the addition of protease inhibitor (i.e., Pefabloc). Before performing the *in vitro* fecal (colonic) fermentations, the absorption of nutrients in the small intestine was simulated by dialyzing the digested infant formulas and human milk. The dialysis is essential to simulate the digestion and absorption of lactose, because the presence of lactose can significantly impact the subsequent *in vitro* (colonic) fermentations. Dialysis was performed using a Pur-A-Lyzer Mega Dialysis Kit purchased from Sigma-Aldrich Chemical Co. (St. Louis, MO, USA) with a molecular weight cut-off of 3.5 kDa. A total of 5 mL of digesta was dialyzed against 5 L of demineralized water for 25 h at room temperature. This resulted in the removal of >95% of the lactose present in the digested samples. The predigested samples were freeze-dried and stored at −20˚C until further usage.

### 2.4. In Vitro Fecal Fermentation

In vitro colonic fermentations were carried out using the CoMiniGut model, as previously reported [22]. In brief, 0.1 M phosphate-buffered saline (PBS), 1 M PBS/20% glycerol (*v*/*v*), and basal colon media (BCM) were prepared and autoclaved at 121 °C for 20 min. Dialyzed digesta of infant formulas with different levels of blocked lysine and human milk were diluted with water and mixed with BCM at a ratio of 1:50. A fecal inoculum of a breast-fed, healthy, term infant (6 months) without antibiotic or probiotic treatments was prepared and used in the *in vitro* fermentations (Ethical Committee of the Capital Region of Denmark; registration number H-20028549). A fresh fecal sample was obtained and homogenized with PBS/20% glycerol (*v*/*v*) at a 1:1 ratio for 120 s using the Stomacher (Stomacher 400; Seward, Worthing, UK), after which glycerol stocks were aliquoted into cryovials and stored at −60 °C before being used as inoculum for the fermentations. The CoMiniGut operates with five parallel reactors with a working volume of 5 mL. Vials were anaerobically inoculated with *in vitro* gut model media, BCM, and fecal inoculum; the model was then run for 24 h under anaerobic conditions, with a physiological temperature of 37 °C (reflecting human body temperature) and incremental pH control throughout the incubation time, from 5.6 to 6.8, to mimic the transition from the proximal to the distal colon. Test conditions were studied in triplicate. After 24 h of fermentation (as a proxy for the colonic passage time), fermenta were collected for gut microbiota and short- and branched-chain fatty acids (SCFAs; BCFAs) analysis. Fermentates were stored at −60 °C before DNA extraction and SCFA measurement.

### 2.5. Determination of Short- and Branched-Chain Fatty Acids (SCFAs/BCFAs)

The concentration of SCFAs and BCFAs in the *in vitro* simulated colonic ferments were determined through gas chromatography–mass spectrometry (GC-MS) according to the method reported by Wiese et al. [22], with minor modifications. Frozen fermenta (200 µL) were thawed and combined with 550 µL of a mixture containing 0.3 M oxalic acid and 20 mM pivalic acid, after which the mixture was centrifuged at 13,000× *g* for 10 min. The supernatant (1 µL injection) with a split ratio of 2:1 was injected into a GC-MS (Agilent 7890A GC and an Agilent 5973 series MSD; Agilent, Waldbronn, Germany) equipped with a Phenomenex Zebron ZB-WAXplus column (30 m × 250 µm × 0.25 µm, Torrance, CA, USA). The GC oven program was set as follows: initial temperature 100 °C, heated to 120 °C at a rate of 10 °C/min, held for 5 min, then ramped to 230 °C at a rate of 40 °C/min before being held at 230 °C for 2 min. Hydrogen was set as the carrier gas with a flow rate of 1.0 mL/min, and the transfer line, ion source, and quadrupole MS temperatures were set to 230, 230, and 150 °C, respectively.

### 2.6. DNA Extraction, 16S rRNA Gene Amplicon Sequencing and Bioinformatics

Fermentation samples were thawed at 4 °C, and 200 µL of the fermenta was used for DNA extraction using the Micro Bead beat AX kit (A&A Biotechnology, Gdansk, Poland), following the manufacturer’s instructions. PCR reactions, library preparation, and purification were conducted as described previously [23]. A negative control using sterile MiliQ water was included during the whole gut microbiota characterization process to identify and remove contaminant sequence reads. Sequencing data generated by an Oxford Nanopore MinION were collected using MinKnow software v19.06.8 (https://nanoporetech.com). The Guppy v3.2.2 basecalling toolkit was used to base call raw fast5 to fastq (https://nanoporetech.com). Porechop v0.2.2 was used for adapter trimming and sample demultiplexing (https://github.com/rrwick/Porechop). Sequences containing quality scores (fastq files) were quality-corrected using NanoFilt (q ≥ 10; read length >1 Kb). Rarefaction curves were generated to evaluate microbial sampling completeness (Supplementary Figure S1). A taxonomy assignment of quality corrected reads using the Greengenes (13.8) database was conducted using the uclast method implemented in parallel_assign_taxonomy_uclust.py Qiime (v1.9.1). The uclust settings were tuned on mock communities (–similarity 0.8; min_consensus_fraction 0.51), assuring annotations at the lowest taxonomic level with no false positive annotations. Data analyses were performed using R packages (v4.0.2). Alpha (Observed species and Shannon diversity index) and beta diversity (based on Bray–Curtis dissimilarity metrics) indices were calculated. Permutational multivariate analysis of variance (PERMANOVA) was used to test differences between categories based on Bray–Curtis dissimilarity metrics. *t*-test was used to assess the alpha diversity indexes and the mean values of SCFAs and BCFAs among different substrates. Spearman was used to assess the correlation between the abundance of the taxa and glycation levels. *p* < 0.05 was regarded as a statistically significant difference.

## 3. Results and Discussion

To investigate whether the level of protein glycation in infant formula could impact infant microbiota composition and activity, we performed an *in vitro* fermentation study using predigested infant formulas with different levels of blocked lysine compared to predigested human milk as a reference.

**Figure 1:**
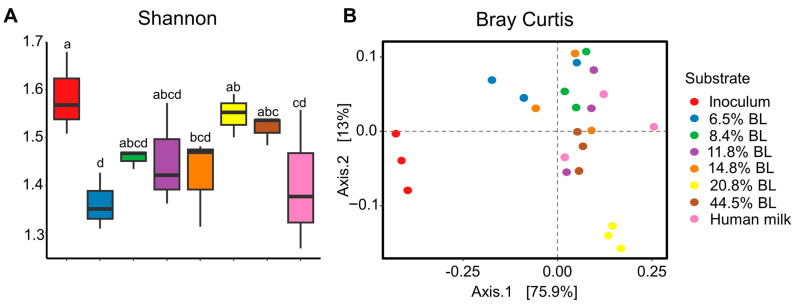
(**A**) Shannon diversity index after 24 h of *in vitro* colon simulated fermentations using infant fecal inoculum. (**B**) Bray-Curtis based dissimilarity metrics based depiction of GM differences between inoculum and after 24 h *in vitro* simulated colonic passage. The percentage of BL represents different levels of blocked lysine in infant formulas. *t*-test was used to assess the alpha diversity index. *p* < 0.05 was regarded as a statistically significant difference and labeled with different letters.

On the basis of the 16S rRNA gene sequencing analysis, the alpha diversity (Shannon index) was shown to increase upon an increase in glycation level in the infant formula (Figure 1). The alpha diversity of the fermentation sample that corresponds to the infant formula with the lowest blocked lysine levels was most similar to the results obtained with the human milk sample. Breast feeding is linked to the optimal development of gut microbiota early in life, which has been associated with a lower incidence of diseases like asthma and childhood obesity [24]. As this trajectory is characterized by a gradual increase in the number of microbiota members, it can be interpreted as positive that the low glycated infant formula sample leads to an *in vitro* simulated gut microbiota Shannon diversity score that is comparable to human milk. No statistical differences were observed with respect to the microbial community composition as determined by Bray–Curtis dissimilarity metrics.

**Figure 2:**
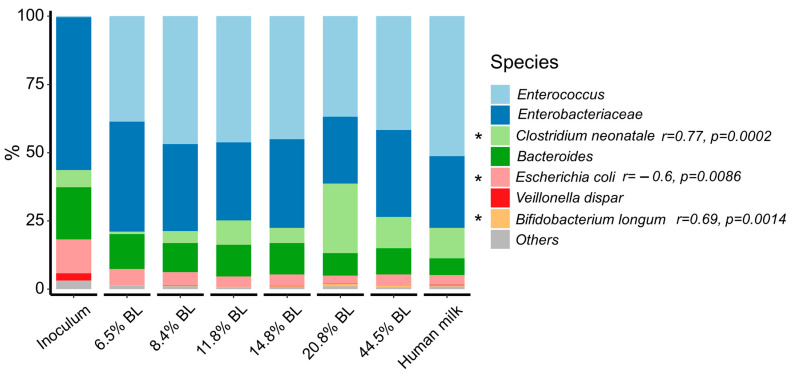
Microbiome composition of *in vitro* simulated colonic fermentations after 24 h, using infant formula with different glycation levels and human milk as substrates. The composition of the initial inoculum is shown for comparison. Species level of the 15 most abundant species. % BL represents the different levels of blocked lysine in the infant formula samples. Spearman correlation test was used to assess the correlation between the abundance of the taxa and glycation levels. Significant correlations (*p* < 0.05) are marked with an asterisk, “r”: correlation coefficient.

In all samples, *Firmicutes*, *Proteobacteria*, *Bacteroidetes,* and *Actinobacteria* constituted the main phyla and the relative abundance of *Firmicutes* increased after 24 h of fermentation. As a reflection of the inoculum, the relative abundance of *Enterobacteriaceae* was relatively high, and that of *Bifidobacterium* was rather low. The relatively high fecal abundance of *Enterobacteriacieae* is not uncommon in infants, with, e.g., Vatanen et al. reporting an, on average, ≈25% relative abundance around the age of 6 months [25]. The changes in the relative abundance of the various microbiota genera and species between the samples were revealed after 24 h of fermentation (Figure 2).

The most discriminating significant difference between samples with different blocked lysine levels constituted the increase in the relative abundance of *Clostridium neonatale* upon an increase in glycation level, especially for the 20,8% blocked lysine, which showed 25.5% of *Clostridium neonatale* as compared to the sample with the lowest level of BL, which only showed 0.99%, mainly at the expense of *Bacteroides. Clostridium neonatale* has been associated with necrotizing enterocolitis (NEC) in preterm neonates [26,27]. While its potential role in the gut of term infants is less described, the bacterium has been linked with gas formation [28], which can cause GI discomfort.

**Figure 3:**
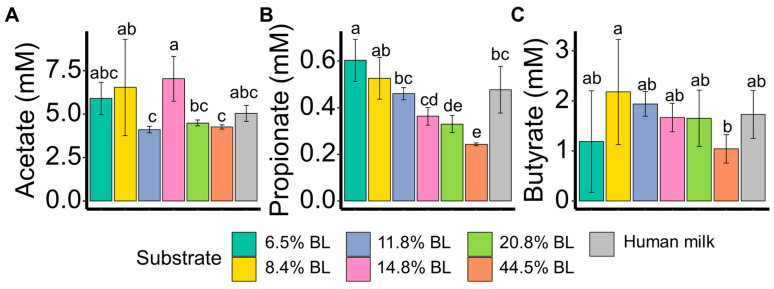
Acetate (**A**), propionate (**B**), and butyrate (**C**) levels (mM) after 24 h fermentation with infant’s feces. % BL represents different levels of blocked lysine in infant formulas. *t*-test was used to compare the mean values of metabolites among the different substrates. *p* < 0.05 was regarded as a statistically significant difference and labeled with different letters.

To reveal the discriminating impact of blocked lysine levels on the microbiota activity, the concentrations of acetate, propionate, butyrate, and the BCFAs iso-butyric acid, iso-valeric acid, and 2-methyl butyric acid after 24 h of *in vitro* simulated colonic fermentation were determined (Figure 3, Supplementary Figure S2). Acetate and butyrate concentrations were shown not to be significantly correlated with blocked lysine levels. For the SCFA propionate, the concentration was found to be inversely correlated to blocked lysine levels. The fermentation samples with human milk, like the less blocked lysine samples (i.e., 8.4, 11.8, and 14.8%), showed higher propionate levels as compared to the highly blocked lysine samples (i.e., 20.8 and 44.5%). For the BCFAs iso-valeric acid, 2-methyl butyric acid, and iso-butyric acid, no significant differences were revealed (Supplementary Figure S2).

Gut microbiota-derived SCFAs are of relevance as they can provide energy to the infant. Colonic propionate is of particular interest as it has been shown to induce short-term appetite regulation [29]. Here, propionate concentrations were shown to be inversely correlated with glycation levels, with the 44.5% blocked lysine sample only giving rise to 0.24 mM propionate (Figure 3B), which was 60% lower as compared to the lowest glycated sample (6.4% blocked lysine). When fermented in the gut, different amino acids can yield different relative proportions of the main SCFAs: acetate, propionate and butyrate [30]. It could be speculated that the glycation of lysine influences the amino metabolization in the *in vitro* simulated infant gut, thereby also influencing propionate levels. In a recent cross-sectional observational study with four commercially available infant formulas, it was shown that infants fed the infant formula with the lowest level of glycated protein were reported to have less crying/fussy time during the night [16]. Interestingly, it has already been hypothesized that propionate can impact infant sleep [31]. A clear association between propionate levels and sleep was observed in infants aged 7 and 8 months, with just a 1% higher proportion of propionate being associated with a 6 min increase in the longest uninterrupted sleep overnight. Also, fecal propionate, as produced by the gut microbiota, was shown to be lower in preschool-aged children who had a longer wake-time after sleep onset (high WASO) [32]. Although the underlying mechanism of the connection with sleep has not yet been revealed, this metabolite could be one of the key mediators connecting the gut microbiota and sleep.

Although the insights were obtained with the fecal material of one infant, a limitation of the study, the findings, which should ideally be replicated in a study with higher statistical power, underscore the importance of producing high-quality, nutritious, and easily digestible infant formula by ensuring the minimal processing of the products during manufacturing. Thus, the beneficial impact of sensitive components that could, indirectly, be exerted through their metabolization by the gut microbiota, e.g., an increase in the production of specific metabolites like propionate, as shown here, can be retained. This not only applies to the nutritional aspects of infant formula but also to its physiological impacts, including GI health and wellbeing and even sleep. With respect to the latter, as this study only applied 16S rRNA gene amplicon sequencings in an ex vivo setting, shotgun metagenomics sequencing could substantiate the findings *in vivo* and provide insights into specific microbiota members and microbial functions/pathways that have the potential to interact with the brain in an intervention study comparing the impact on gut microbiota composition and the activity of commercial formulas with discriminating BL levels.

## 4. Conclusions

In summary, this study found that higher protein glycation levels in infant formula increase microbial diversity and the relative abundance of *Clostridium neonatale* but decreased propionate production during a 24 h *in vitro* simulated colonic fermentation passage. Our results provide leads for further in-depth research investigating the influence of protein glycation of infant formula on metabolite production and the resulting health implications for infants.

## Data Availability

The data are not publicly available due to privacy. The data supporting the findings of this study are available from the corresponding author [A.N.] upon reasonable request.

## Supplementary Materials

The following supporting information can be downloaded at: https://www.mdpi.com/article/10.3390/nu16234047/s1, Figure S1: Rarefaction curves (species level) for the in vitro simulated colon fermentations, Figure S2: Iso-butyrate (A), iso-valerate (B), and 2-methylbutyrate (C) production after 24 h fermentation with infant’s feces. The percentage of BL represents the different levels of blocked lysine in infant formulas. *t*-test was used to compare the mean values of metabolites among different substrates. *p* < 0.05 was regarded as a statistically significant difference and labeled with different letters.
