# Supplementary material for: Propionate Production by Infant Fecal Microbiota Is Inversely Correlated with the Protein Glycation Level of Supplemented Infant Formula Ex Vivo
Source: Nutrients. 2024 Nov 26;16(23):4047. doi: 10.3390/nu16234047 (PMC11643526; doi:10.3390/nu16234047)
Supplement: Supplementary file 1 [file nutrients-16-04047-s001.zip › nutrients-3268948-supplementary.pdf]

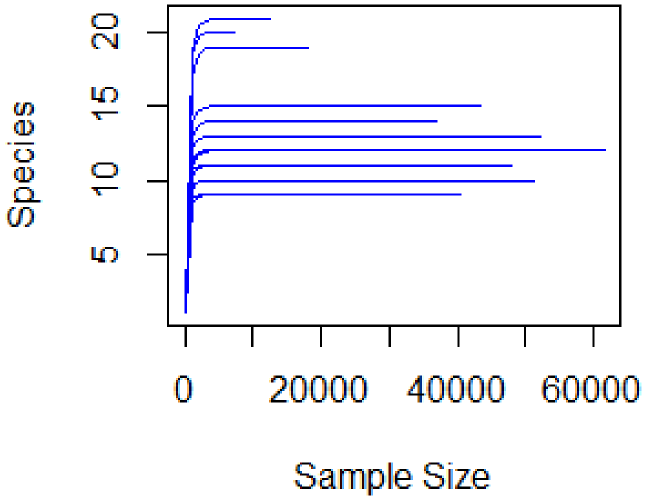

Figure S1. Rarefaction curves (species level) for the *in vitro* simulated colon fermentations.

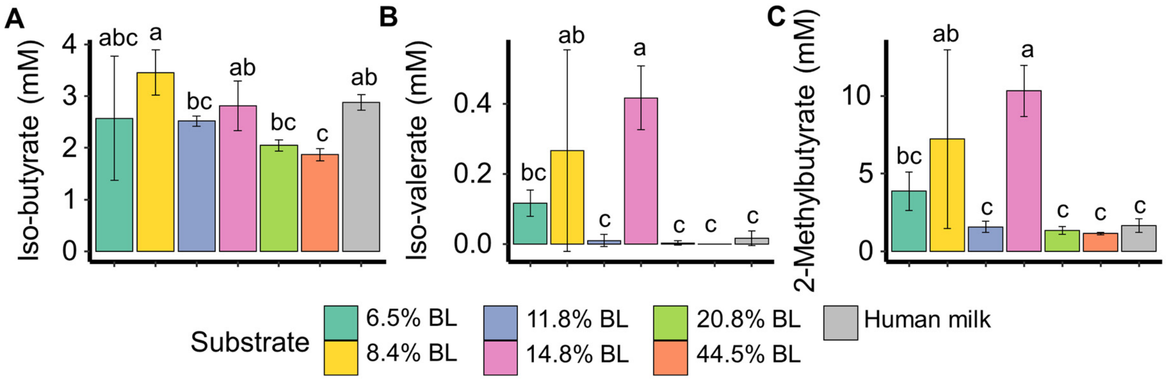

Figure S2: Iso-butyrate (**A**), iso-valerate (**B**), and 2-methylbutyrate (**C**) production after 24 h fermentation with infant's feces. The percentage of BL represents the different levels of blocked lysine in infant formulas. *t*-test was used to compare the mean values of metabolites among different substrates. $p < 0.05$ was regarded as a statistically significant difference and labeled with different letters.
